# Orai/CRACM1 and K_Ca_3.1 ion channels interact in the human lung mast cell plasma membrane

**DOI:** 10.1186/s12964-015-0112-z

**Published:** 2015-07-16

**Authors:** S. Mark Duffy, Ian Ashmole, Dawn T. Smallwood, Mark L. Leyland, Peter Bradding

**Affiliations:** Department of Infection, Immunity and Inflammation, Institute for Lung Health, University of Leicester, Glenfield Hospital, Leicester, LE3 9QP UK; Department of Biochemistry, Henry Wellcome Building, University of Leicester, Lancaster Road, Leicester, LE1 9HN UK; Department of Respiratory Medicine, Glenfield Hospital, Groby Rd, Leicester, LE3 9QP UK

**Keywords:** Orai, K_Ca_3.1, Mast cell

## Abstract

**Background:**

Orai/CRACM1 ion channels provide the major Ca^2+^ influx pathway for FcεRI-dependent human lung mast cell (HLMC) mediator release. The Ca^2+^-activated K^+^ channel K_Ca_3.1 modulates Ca^2+^ influx and the secretory response through hyperpolarisation of the plasma membrane. We hypothesised that there is a close functional and spatiotemporal interaction between these Ca^2+^- and K^+^-selective channels.

**Results:**

Activation of FcεRI-dependent HLMC K_Ca_3.1 currents was dependent on the presence of extracellular Ca^2+^, and attenuated in the presence of the selective Orai blocker GSK-7975A. Currents elicited by the K_Ca_3.1 opener 1-EBIO were also attenuated by GSK-7975A. The Orai1 E106Q dominant-negative mutant ablated 1-EBIO and FcεRI-dependent K_Ca_3.1 currents in HLMCs. Orai1 but not Orai2 was shown to co-immunoprecipitate with K_Ca_3.1 when overexpressed in HEK293 cells, and Orai1 and K_Ca_3.1 were seen to co-localise in the HEK293 plasma membrane using confocal microscopy.

**Conclusion:**

K_Ca_3.1 activation in HLMCs is highly dependent on Ca^2+^ influx through Orai1 channels, mediated via a close spatiotemporal interaction between the two channels.

## Background

The aberrant activation of mast cells via the high affinity IgE receptor FcεRI results in the release of preformed granule-derived mediators such as histamine and tryptase, the synthesis and release of lipid mediators such as leukotriene (LT)C_4_ and prostaglandin (PG)D_2_, and the generation of numerous cytokines, including IL-4, IL-5 and IL-13 [1]. This process contributes to the pathophysiology of allergic diseases such as asthma, rhinitis, urticaria and anaphylaxis [1].

The influx of extracellular Ca^2+^ is an essential requirement for the FcεRI-dependent release of mast cell mediators [2]. Members of the recently discovered Orai (also known as CRACM) ion channel family provide the major pathway for this influx in both rodent and human lung mast cells (HLMCs) [3–5]. These channels carry Ca^2+^ selective currents (I_CRAC_) that are activated when endoplasmic reticulum (ER) Ca^2+^ stores are emptied. The family contains three members Orai1, Orai2 and Orai3 [6, 7]. They show a high degree of sequence homology but have distinct functional properties [6, 7]. The channel pore is contained within the Orai channel subunit [8–11]_._ An ER membrane protein, STIM1, acts as the sensor of the ER Ca^2+^ concentration and transmits this information to the channel pore [12]. Human and rodent mast cells express all three Orai subunits at the mRNA level, although Orai1 is the dominant channel which is activated following cross-linking of FcεRI [3–5]. In HLMCs pharmacological block of Orai channels reduces Ca^2+^ influx, degranulation, LTC_4_ release and cytokine secretion [3]. Similarly, Ca^2+^ influx, degranulation, LTC_4_ release and TNFα production are all greatly reduced in foetal liver-derived mast cells from an Orai1 knockout mouse [4].

Both human and rodent mast cells also express functional intermediate conductance Ca^2+^ activated K^+^ channels (K_Ca_3.1) [13–15]. In human mast cells K_Ca_3.1 constitutes the major K^+^ selective conductance [13, 14]. K_Ca_3.1 channels are activated in mast cells by a rise in the cytosolic free Ca^2+^ concentration following FcεRI-dependent activation. Calmodulin, which is tightly bound near the C-terminus of the K_Ca_3.1subunit, acts as the Ca^2+^ sensor. The key consequence of K_Ca_3.1 opening during cell activation is the hyperpolarisation of the cell membrane. This maintains the electrical driving force for Ca^2+^ influx through store operated channels such as Orai, and enhances Ca^2+^ influx through Orai channels because they are strongly inwardly rectifying and therefore conduct larger currents at negative membrane potentials [16]. Thus, activation of K_Ca_3.1 channels using the K_Ca_3.1 channel opener 1-ethyl-2-benzimidazolinone (1-EBIO) enhanced IgE-dependent Ca^2+^ influx and degranulation in HLMCs [14]. In contrast IgE-dependent Ca^2+^ influx and degranulation was significantly reduced in bone-marrow derived mast cells isolated from a K_Ca_3.1 knockout mouse [15]. K_Ca_3.1 has also been shown to be important for HLMC migration [17].

K_Ca_3.1 channel activity disappears when extracellular Ca^2+^ is removed from cultured human mast cells that have been activated through FcεRI [13], and channel activity is not induced by several stimuli that release Ca^2+^ from intracellular stores but which do not stimulate Ca^2+^ influx [17]. In addition, K_Ca_3.1 currents were not elicited in HLMCs activated by the Ca^2+^ ionophore A23187 [13]. This suggests that the activation of K_Ca_3.1 channels by Ca^2+^ under physiological conditions relies on a tight spatiotemporal Ca^2+^ signal provide by Ca^2+^ influx channels. We therefore hypothesised that there is a close functional relationship between K_Ca_3.1 and Orai channels in HLMCs, and that the two channels may interact physically to maintain tight spatiotemporal control of their activity. To test this hypothesis we have examined the effects of Orai channel inhibition in HLMCs on K_Ca_3.1 channel activity and used co-immunoprecipitation and confocal microscopy to examine the physical interaction between K_Ca_3.1 and Orai channels in co-transfected HEK293 cells.

## Results

### K_Ca_3.1 requires extracellular Ca^2+^ for activation by FcεRI cross-linking in HLMCs

HLMC activation via FcεRI stimulates the release of Ca^2+^ from intracellular stores followed by the influx of extracellular Ca^2+^ through Orai channels. Influx of extracellular Ca^2+^ is essential for HLMC degranulation and lipid mediator synthesis [2].

**Fig. 1:**
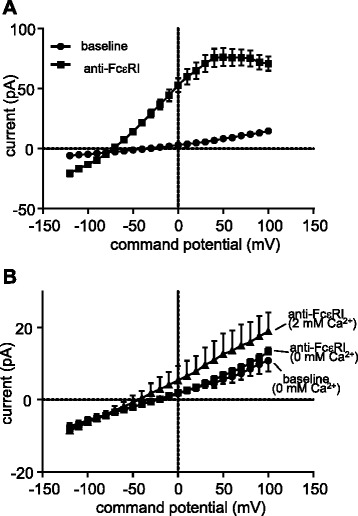
FcεRI-dependent HLMC K_Ca_3.1 currents require the presence of external Ca^2+^. **a** Current–voltage curves from HLMCs activated via FcεRI in the presence external Ca^2+^ (2 mM). **b** Current–voltage curves from HLMCs activated via FcεRI in the absence of external Ca^2+^. Typical K_Ca_3.1 currents appear in the presence of Ca^2+^ (2 mM) but not when it is absent. Small K_Ca_3.1 currents appear in (**b**) when Ca^2+^ is then introduced. Data presented as mean ± SEM

We showed previously that K_Ca_3.1 currents elicited in human mast cells by FcεRI-dependent activation disappear on removal of extracellular Ca^2+^, demonstrating the need for extracellular Ca^2+^ influx for the *maintenance* of K_Ca_3.1 activity [13]. Here, activation of HLMCs by cross-linking FcεRI in the presence of extracellular Ca^2+^ evoked typical K_Ca_3.1 currents as previously described in 81 % of cells studied, with a mean whole cell current at +40 mV in responding cells increasing from 6.8 ± 0.8 pA (reversal potential −31.4 ± 3.2 mV) at baseline to 75.6 ± 7.6 pA post anti-FcεRI (reversal potential −72.0 ± 9.3 mV) (*n* = 27 cells) (*p* < 0.0001 and *p* < 0.0001 for current and reversal potential respectively compared to baseline) (Fig. 1a). In contrast, in the absence of extracellular Ca^2+^, no K_Ca_3.1 currents developed:current at +40 mV pre and post anti-FcεRI 4.5 ± 1.2 pA and 5.5 ± 1.1 pA respectively (*p* = 0.42), reversal potential pre and post anti-FcεRI −24.2 ± 1.8 mV and −22.0 ± 2.0 mV respectively (*p* = 0.71)(*n* = 9 cells)(Fig. 1b). Subsequent addition of extracellular Ca^2+^ (2 mM) to these cells following FcεRI-dependent activation induced only small K_Ca_3.1 currents (11.0 ± 5.4 pA at +40 mV, *p* = 0.017 compared to no Ca^2+^) and a significant shift in reversal potential (−43.8 ± 7.4 mV, *p* = 0.015 compared to no Ca^2+^)(Fig. 1b). The small K_Ca_3.1 currents seen here on subsequent addition Ca^2+^ here are in keeping with the known desensitisation of signalling pathways that occur when FcεRI is activated in the absence of extracellular Ca^2+^ [18, 19].

In summary, Ca^2+^ influx from the extracellular fluid is a critical requirement for the *initial* opening of K_Ca_3.1 after FcεRI-dependent activation in HLMCs, as well as its *maintenance* following activation as described previously [13].

### Orai channel block attenuates K_Ca_3.1 activation

**Fig. 2:**
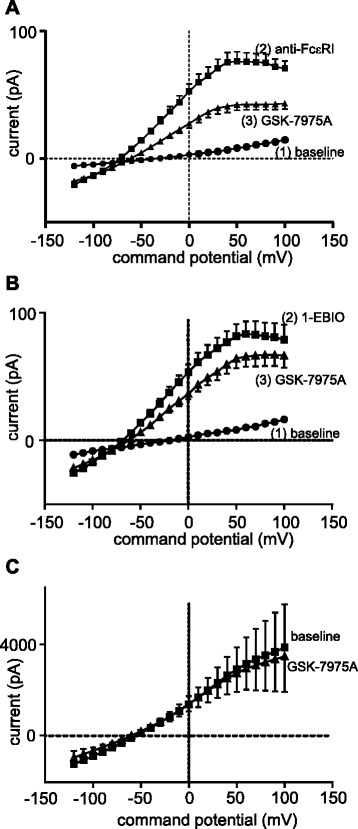
FcεRI- and 1-EBIO-dependent HLMC K_Ca_3.1 currents are attenuated by an Orai channel blocker. **a** K_Ca_3.1 currents induced following FcεRI-dependent activation are attenuated in HLMCs following addition of the Orai channel blocker GSK-7975A (*n* = 27 cells). **b** K_Ca_3.1 currents induced following 1-EBIO-dependent activation are attenuated following addition of the Orai channel blocker GSK-7975A (*n* = 29 cells). Data presented as mean ± SEM. **c** Overexpressed K_Ca_3.1 channels in HLMCs were constitutively active and were not blocked by GSK-7975A (1 μM)(*n* = 4 cells, *p* = 0.43 at +40 mV)

To assess whether Ca^2+^ influx through Orai channels contributes to K_Ca_3.1 opening, we used the selective Orai channel blocker, GSK-7975A (a kind gift GlaxoSmithKline) [3, 20]. K_Ca_3.1 currents elicited in response to FcεRI-dependent HLMC activation were significantly attenuated with the subsequent addition of 1 μM GSK-7975A, a concentration that suppresses Orai currents by >90 % in our hands [3] (Fig. 2a). Thus baseline whole cell currents of 6.8 ± 0.8 pA at +40 mV increased to 75.6 ± 7.6 pA post anti-FcεRI and were reduced to 40.9 ± 4.7 pA following addition of GSK-7975A (*p* < 0.0001, *n* = 27 cells). A small but significant positive shift in reversal potential was also evident with the addition of GSK-7975A (post anti-FcεRI −72.0 ± 9.3 mV, post GSK-7975A −61.3 ± 6.7 mV, *p* = 0.009) (Fig. 2a).

1-EBIO opens K_Ca_3.1 by enhancing its sensitivity to [Ca^2+^]_i_. Thus at 100 μM EBIO, maximal K^+^ currents are achieved in the presence of 100 nM free Ca^2+ ^[21], which is below the resting [Ca^2+^]_i_ of most cell types including HLMCs [14]. Interestingly, 1-EBIO-dependent K_Ca_3.1 currents were also attenuated although to a lesser degree by GSK-7975A (Fig. 2b). Thus baseline whole cell currents of 7.4 ± 0.8 pA at +40 mV increased to 76.8 ± 8.8 pA post 1-EBIO, and were reduced to 59.4 ± 7.2 pA following addition of GSK-7975A (*p* < 0.0001, *n* = 29 cells). The reduction in K_Ca_3.1 current induced by GSK-7975A following FcεRI-dependent HLMC activation was significantly greater than following 1-EBIO-dependent K_Ca_3.1 activation (*p* = 0.039).

To confirm that GSK-7975A does not directly block K_Ca_3.1 currents, GFP-K_Ca_3.1 was overexpressed in HLMCs. This generated large (nA) constitutively active K_Ca_3.1 currents that were not blocked by GSK-7975A (1 μM)(Fig. 2c).

### The Orai1 E106Q dominant-negative mutant ablates K_Ca_3.1 currents

**Fig. 3:**
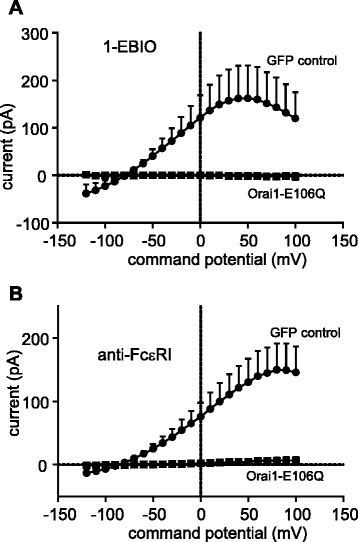
FcεRI- and 1-EBIO-dependent HLMC K_Ca_3.1 currents are inhibited by expression of an Orai1-E106Q dominant-negative mutant. Transduction of HLMCs with an Orai1-E106Q dominant-negative mutant ablated (**a**) 1-EBIO- dependent and (**b**) FcεRI-dependent K_Ca_3.1 currents. For clarity, data are presented as the subtracted net activation-dependent currents (activation minus baseline) for each condition, expressed as mean ± SEM

To further investigate the role of Orai channels in the regulation of K_Ca_3.1 in HLMCs, the effect of a dominant-negative mutant of Orai1 (E106Q) was assessed [5]. While 7/7 GFP-transduced control HLMCs expressed robust K_Ca_3.1 currents following exposure to 1-EBIO (net current at +40 mV 40.9 ± 19.5 pA, *n* = 7 cells), no K_Ca_3.1 currents could be elicited in cells transduced with Orai1-E106Q (net current at +40 mV −1.1 pA, *n* = 6 cells, *p* = 0.04 compared to GFP) (Fig. 3a and b).

Similarly 8/8 GFP-transduced control HLMCs expressed robust K_Ca_3.1 currents following exposure to anti-FcεRI (net current at +40 mV 121.6 ± 34.2 pA, *n* = 8 cells), but no FcεRI-dependent K_Ca_3.1 currents could be elicited in cells transduced with Orai1-E106Q (net current at +40 mV 4.1 ± 3.2 pA, *n* = 9 cells, *p* = 0.0023 compared to GFP)(Fig. 3b).

### Orai1 but not Orai2 co-immunoprecipitates with K_Ca_3.1

**Fig. 4:**
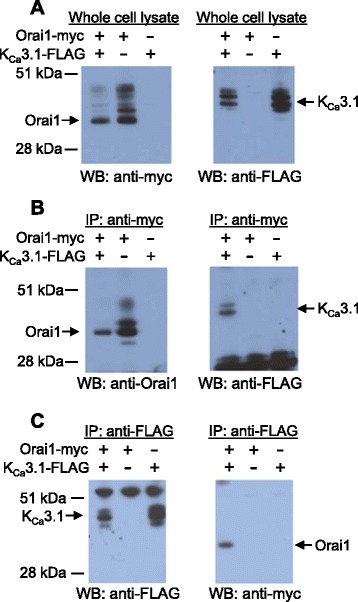
Orai1 and K_Ca_3.1 proteins co-immunoprecipitate. **a** Western blots using either an antibody recognising the myc epitope (left) or an antibody recognising the FLAG epitope (right) of HEK293 cell lysates. Lysates expressed either myc epitope tagged Orai1, FLAG epitope-tagged K_Ca_3.1, or both as indicated in the panel above. **b** Lysates of HEK293 cells expressing the indicated proteins were immunoprecipitated with an anti-myc antibody. Immunoprecipitates were then Western blotted using either an anti-Orai1 antibody (left) or an anti-FLAG antibody (right). **c** As (**b**) except cell lysates were immunoprecipitated with an anti-FLAG antibody and then Western blotted with an anti-FLAG antibody (left) or an anti-myc antibody (right). Blots shown are representative of 3 independent experiments

The proposed functional interaction between Orai and K_Ca_3.1 channels in HLMCs led us to investigate whether these channels interact physically. We therefore expressed myc epitope-tagged Orai1 and Orai2 and FLAG epitope-tagged K_Ca_3.1 in HEK293 cells and tested for potential interactions by co-immunoprecipitation. Expression of epitope tagged channels was confirmed by Western blotting using antibodies raised against the appropriate epitope tag (Fig. 4a). Multiple bands were observed on blotting for K_Ca_3.1-FLAG protein using an anti-FLAG antibody, with band sizes of 48 kDa (the predicted size of K_Ca_3.1), and less. A similar band pattern has recently been observed on Western blotting for K_Ca_3.1 in human fibrocytes, lung fibroblasts and airway smooth muscle cells, and may reflect differential splicing modification [22–24]. When an anti c-myc antibody was used to immunoprecipitate Orai1-myc protein, K_Ca_3.1-FLAG was found to be co-immunoprecipitated (Fig. 4b). Co-immunoprecipitation of K_Ca_3.1-FLAG was observed only from lysates of cells expressing both Orai1-myc and K_Ca_3.1-FLAG proteins. Similarly when an anti-FLAG antibody was used to immunoprecipitate K_Ca_3.1-FLAG protein, Orai1-myc protein was co-immunoprecipitated (Fig. 4c). Again co-immunoprecipitation was dependent on co-expression of both proteins in HEK293 cells.

**Fig. 5:**
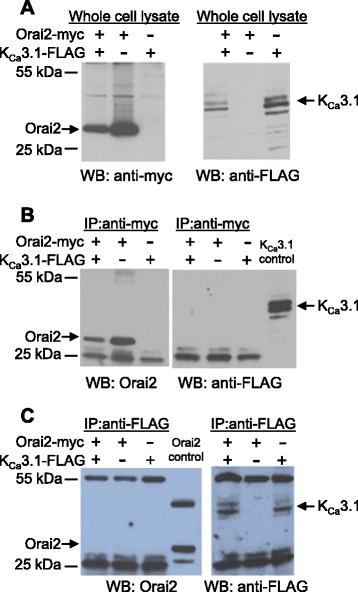
Orai2 and K_Ca_3.1 proteins do not co-immunoprecipitate under the conditions used to co-immunoprecipitate Orai1 and K_Ca_3.1. **a** Western blots using either an antibody recognising the myc epitope (left) or an antibody recognising the FLAG epitope (right) of HEK293 cell lysates. Lysates expressed either myc epitope tagged Orai2, FLAG epitope-tagged K_Ca_3.1, or both as indicated in the panel above. **b** HEK293 cell lysates expressing the proteins indicated in the panel above were immunoprecipitated with an anti-myc antibody. Immunoprecipitates were then Western blotted using either an anti-Orai2 antibody (left) or an anti-FLAG antibody (right). Control HEK293 cell lysate expressing K_Ca_3.1-FLAG protein. **c** As (**b**) except cell lysates were immunoprecipitated with an anti-FLAG antibody and then Western blotted with an anti-Orai2 antibody (left) or an anti-FLAG antibody (right). Control HEK293 cell lysate expressing Orai2-myc protein. Blots shown are representative of 3 independent experiments

In contrast, under identical reaction conditions, no co-immunoprecipitation of K_Ca_3.1-FLAG protein was observed when the anti c-myc antibody was used to immunoprecipitate Orai2-myc protein (Fig. 5a and b). Similarly in the reverse experiment using the anti-FLAG antibody to immunoprecipitate K_Ca_3.1-FLAG, no Orai2-myc protein was co-immunoprecipitated (Fig. 5c). We were unable to test for Orai3-myc and K_Ca_3.1-FLAG co-immunoprecipitation, since we were unable to satisfactorily demonstrate co-expression of Orai3-myc and K_Ca_3.1-FLAG protein in HEK293 cells (data not shown).

### Orai1 and K_Ca_3.1 co-localise in the plasma membrane of HEK-293 cells

**Fig. 6:**
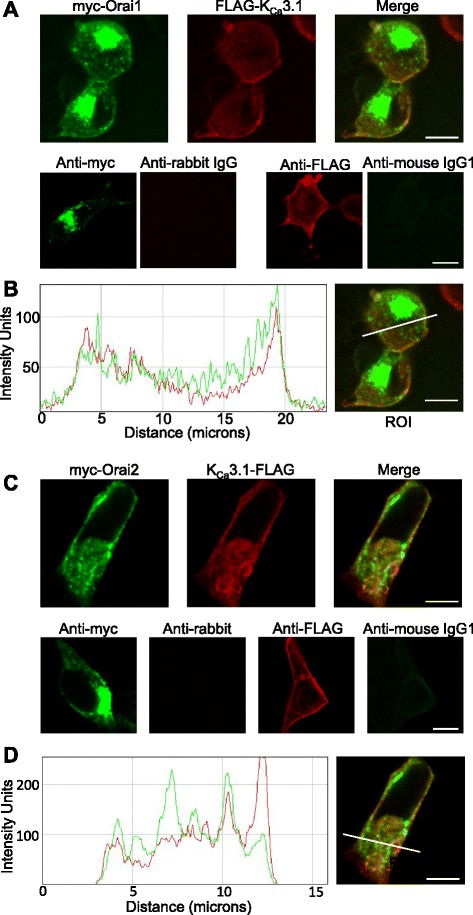
Orai1 and K_Ca_3.1 co-localise in the plasma membrane. **a** HEK293 cells, dually transfected with FLAG-tagged K_Ca_3.1 and myc-tagged Orai1 and then immunostained, show co-localisation in the plasma membrane by single plane confocal microscopy (top panels). Dually transfected HEK293 show negative staining for appropriate isotype controls (bottom panels): rabbit IgG control, dual stained with anti-myc, and mouse IgG1 control dual stained with anti-FLAG. **b** Fluorescence intensity plot shows increased fluorescence at the plasma membrane. myc-Orai1 is shown in green and FLAG-K_Ca_3.1 in red. Arrows indicate increased fluorescence where the region of interest (ROI) intersects the plasma membrane. **c** HEK293 cells, dually transfected with FLAG-tagged K_Ca_3.1 and myc-tagged Orai2 and then immunostained, show poor co-localisation in the plasma membrane by single plane confocal microscopy (top panels). Dually transfected HEK293 show negative staining for appropriate isotype controls (bottom panels): rabbit IgG control, dual stained with anti-myc, and mouse IgG1 control dual stained with anti-FLAG. **d** Fluorescence intensity plot shows poor co-localisation of K_Ca_3.1 and Orai2 signals. myc-Orai2 is shown in green and FLAG-K_Ca_3.1 in red. Scale bars are 10 μm

To assess whether Orai1 and K_Ca_3.1 co-localise in plasma membranes, we dually transfected HEK-293 with FLAG-tagged K_Ca_3.1and myc-tagged Orai1 or Orai2 (HLMCs cannot be transfected efficiently). Subsequent analysis of the immunostained tags using confocal microscopy showed clear co-localisation of Orai1 and K_Ca_3.1 in the HEK293 plasma membrane (Fig. 6a and b), but minimal co-localisation of K_Ca_3.1 with Orai2 (Fig. 6c and d).

## Discussion

The release of mast cell mediators following cell activation requires the influx of extracellular Ca^2+^. We and others have shown that members of the Orai channel family play a major role in this Ca^2+^ influx in both human and rodent mast cells [3, 4]. The intermediate conductance Ca^2+^ activated K^+^ channel K_Ca_3.1 is also a key regulator of Ca^2+^ influx into mast cells [13–15], as activated K_Ca_3.1 channels maintain the driving force for Ca^2+^ entry by hyperpolarising the plasma membrane. Here we show for the first time that K_Ca_3.1 activation has an obligate dependency on Ca^2+^ influx through Orai1 channels, which appears to be mediated via an intimate spatiotemporal interaction.

We have demonstrated previously that K_Ca_3.1 opens in HLMCs following FcεRI-dependent activation, disappears on removal of extracellular Ca^2+^, and does not open after stimulation with other agonists which globally raise intracellular Ca^2+^ in HLMCs such as chemokines, PAF, LPA and the Ca^2+^ ionophore A23187 [13, 17, 25]. This suggested that tight Ca^2+^ microdomains regulate K_Ca_3.1 activity. Here we found that activation of FcεRI-dependent K_Ca_3.1 currents required the presence of extracellular Ca^2+^, were attenuated by a selective Orai channel blocker, and were ablated by transduction of a dominant-negative Orai1 mutant. This implies that Ca^2+^ engages K_Ca_3.1 at the point of entry through Orai channels. The ability of K_Ca_3.1 to co-immunoprecipitate and co-localise with Orai1, the dominant Orai channel in HLMCs, suggests the presence of a tight Orai1-K_Ca_3.1 signalplex in the HLMC plasma membrane.

It is perhaps surprising that Orai1 interacted physically with K_Ca_3.1 but Orai2 did not. However, Orai1 contains a C-terminal SHYA sequence which may act as a PDZ binding site. Although it does not conform to the class II or class III binding sites which are characterised by X-Hydrophobic-X-Hydrophobic or X-Neg charged-X-V/I/A, there is variation and other sequences will bind as well. If this is a PDZ binding site, then there is the potential for K_Ca_3.1 and Orai1 to form part of a macromolecular complex organised by PDZ-containing scaffolding proteins. Orai1also interacts with TRPC family channels via a direct physical interaction at the N- and C-termini, but this is also true for Orai2 and Orai3 [26], so it seems unlikely that this is the mechanism mediating the interaction of Orai1 with K_Ca_3.1. However, the N-terminus of Orai1 but not Orai3 forms a membrane-delimited signalplex with NFAT via the scaffolding protein AKAP79 [27]. Further work will be required to establish the exact mechanism of the K_Ca_3.1-Orai1 interaction.

Orai1 currents are only activated following store depletion by STIM1, and our data do not directly address whether STIM1 plays a role in the co-regulation of Orai1 and K_Ca_3.1. However, the co-immunoprecipitation and confocal imaging experiments were performed in resting cells following over-expression, while the functional interaction is evident in lung mast cells following IgE-dependent activation, so it is likely that the interaction occurs independently of Ca^2+^ store depletion. This would be in keeping with previous studies showing that another Ca^2+^ activated K^+^ channel, K_Ca_1.1 (bK_Ca_), forms a signalling complex consisting of the β_2_-adrenoreceptor, the L-type calcium channel Ca_v_1.2 and the A-kinase anchoring proteins AKAP79 and AKAP150 [28]. The Ca^2+^required for activation of K_Ca_1.1 is provided by voltage-gated Ca^2+^ channels, either Ca_v_1.2, Ca_v_2.1 or Ca_v_2.2 [29]. There is also limited evidence that K_Ca_3.1 may form a signalplex with the β_2_-adrenoreceptor [30], so it will be interesting to assess whether a similar complex utilises K_Ca_3.1 and Orai1.

A close association of K_Ca_1.1 with voltage-gated Ca^2+^ channels is thought to be necessary because activation requires a relatively high intracellular Ca^2+^ concentration of 10 μM or more. Such concentrations are thought only to be found in so called Ca^2+^ nanodomains in the immediate vicinity of the open Ca_v_ channels. In contrast, K_Ca_3.1 has a far higher affinity for Ca^2+^, with a value for half maximal activation (EC_50_) of approximately 0.3 μM [31]. This high affinity has been considered to obviate the requirement for a close interaction with a Ca^2+^ source [32]. However, it is clear that if K_Ca_3.1 is coupled closely to a particular Ca^2+^ entry pathway and signalplex, there will be greater sensitivity and specificity with regards to K_Ca_3.1 channel activation. Furthermore, Orai channels have a very low single channel conductance, estimated to be more than 100-fold lower than for voltage-gated Ca^2+^ channels [33, 34]. As a consequence the increase in Ca^2+^ concentration in the vicinity of an open Orai channel is likely to be relatively small. In mast cells the Ca^2+^ concentration has been estimated to be of the order of 2 μM at a distance of 10 nm from an open Orai channel, falling steeply with increasing distance [35]. While clustering of (open) Orai channels in mast cells would lead to considerably higher local Ca^2+^concentrations, a close association of Orai1 with K_Ca_3.1 in mast cells may therefore be required to ensure efficient activation of K_Ca_3.1 channels and so regulation of Ca^2+^ influx.

The importance of Ca^2+^ microdomains for the regulation of specific Ca^2+^-dependent cell responses was highlighted recently by Parekh [35, 36]. For example there is evidence of distinct Orai channel signalling domains regulating the activity plasma membrane-associated enzymes such as Syk and cPLA2, and transcription factors such as NFAT [27, 37, 38]. A close K_Ca_3.1-Orai1 interaction would therefore appear to make sense from several perspectives. The activation of K_Ca_3.1 independently of global intracellular Ca^2+^ will fine-tune signals requiring Orai1 activation, with rapid and selective feedback ensuring that the Ca^2+^ signal is maintained, supporting Ca^2+^-dependent local membrane-associated signalling events. Furthermore, modulation of K_Ca_3.1 by de-phosphorylation for example [39], or β_2_-adrenoceptor signalling [30], has the potential to feedback on Orai1 and limit Ca^2+^ entry. In resting cells where K_Ca_3.1 and perhaps Orai1 expression may be relatively low [40], their co-localisation would ensure that efficient signalling could occur during an initial cell response. In addition, in cells expressing numerous K_Ca_3.1 and Orai channels, there would be enhanced sensitivity to low grade stimuli, thus increasing the dynamic range, specificity and fidelity of the response to an external stimulus. It is also evident that K_Ca_3.1 and Orai channels are localised to specific regions of activated cells. For example, localised K_Ca_3.1 channel activity regulates shrinkage of the uropod in migrating cells even although the channels are distributed throughout the plasma membrane [41], while both Orai1 and K_Ca_3.1 co-localise at the immunological synapse in activated T cells [42, 43]. Their ability to form a close physical interaction would ensure that they interact specifically in these regions. It is also clear that Orai1-3 have distinct physiological roles and electrophysiological characteristics [6, 44, 45]. A selective interaction between Orai1 and K_Ca_3.1 would ensure that unwanted K_Ca_3.1 activation does not occur in Orai2 or Orai3-dependent cell responses. The physiological relevance of this interpretation is supported by previous work in HLMCs. These cells express both Orai1 and Orai2 protein, but FcεRI-dependent degranulation and leukotriene release is driven largely by Orai1 [5].

## Conclusions

There is a close spatio-temporal and functional interaction between K_Ca_3.1 channels and Orai1 channels in HLMCs with evidence of a physical interaction leading to a restricted membrane-delimited signalplex. This is likely to facilitate the selective activation and fine-tuning of K_Ca_3.1-Orai1-dependent cell processes.

## Methods

### Human mast cell purification and cell culture

HLMCs were purified from enzymatically dispersed healthy human lung obtained within one hour of surgery for lung cancer [46, 47]. Cells were cultured in Dulbecco’s modified Eagle’s medium (DMEM, Invitrogen) containing 10 % foetal calf serum and 100 ng/ml stem cell factor, 50 ng/ml IL-6 and 10 ng/ml IL-10 as previously described [47]. All human subjects donating lung tissue gave written informed consent and the study was approved by the Leicestershire Research Ethics Committee. Final mast cell purity was >99 %.

HEK293 cells were cultured in DMEM containing 10 % FCS.

### Orai and K_Ca_3.1 protein expression and co-immunoprecipitation

The construction of vectors directing the expression of c-Myc epitope-tagged Orai1 and Orai2 has been described previously [3]. Briefly full length Orai1 and −2 cDNAs were cloned in frame immediately following the c-Myc epitope tag in vector pCruz Myc (Santa Cruz Biotechnology Inc). For the expression of FLAG epitope tagged K_Ca_3.1, a PCR fragment containing the entire human K_Ca_3.1open reading frame (ORF), an EcoR1 site immediately adjacent to the ATG initiation codon and an Xho1 site immediately adjacent to the stop codon was cloned into the EcoR1/Xho1 sites of vector pcDNA3 (Invitrogen). The stop codon was then removed by site directed mutagenesis. Oligonucleotides 5′ TCGAGGACTACAAAGACGATGACGACAAGTAGC 3′ and 5′ TCGAGCTACTTGTCGTCATCGTCTTTGTAGTCC 3′ encoding the FLAG eiptope (DYKDDDDK) were annealed together and inserted at the Xho1 site of the mutant K_Ca_3.1 construct. The resulting vector directs the expression of K_Ca_3.1 with a FLAG epitope in frame with the last codon of the K_Ca_3.1 ORF. The construct was verified by DNA sequencing.

HEK293 cells were transiently transfected with vectors directing the expression of c-Myc epitope tagged Orai1 and −2 and/or FLAG epitope-tagged K_Ca_3.1 using GeneJuice transfection reagent (Merck Bioscience Ltd). Cells were harvested for lysis 24 h later and lysed in a buffer containing 50 mM Tris pH8.0, 150 mM NaCl, 1 % Triton X-100 and a protease inhibitor cocktail (Sigma). Lysates were centrifuged at 13,200 rpm for 15 min at 4 °C and supernatants used for co-immunoprecipitation experiments.

For the immunoprecipitation of Orai1 or Orai2, supernatants were pre-cleared with protein G-sepharose beads and then incubated with a mouse monoclonal anti-c-Myc antibody (clone 9E10, Sigma) for 16–18 h at 4 °C. Immune complexes were recovered by incubation with protein G-sepharose beads for 15 min at 4 °C and washed 5 times with lysis buffer. Immunoprecipitation of K_Ca_3.1 was as above except rabbit polyclonal anti-FLAG antibody (Sigma) and protein A-agarose were used.

Proteins were separated on 12 % Bis-Tris Nu-Page gels (Invitrogen) and then blotted onto polyvinylidene fluoride membranes. Membranes were blocked with 5 % nonfat milk in phosphate buffered saline. Blots were then probed with rabbit polyclonal antibodies recognising either Orai1 or Orai2 (both Alomone Labs Ltd) [3], anti-c-Myc antibody and/or anti-FLAG antibody as required. Blots were subsequently probed with Clean-Blot IP-horseradish peroxidase conjugate (Thermo Scientific). Immunoreactive bands were visualised using Pierce ECL Western Blotting Substrate (Fisher Scientific, Loughborough, United Kingdom).

HLMCs were transduced with adenoviruses expressing Orai E106Q and GFP control as described previously [5]. HLMCs were also transduced with GFP-K_Ca_3.1 using the same methodology.

### Patch clamp electrophysiology

The whole-cell variant of the patch-clamp technique was used [3, 13]. Patch pipettes were made from borosilicate fibre-containing glass (Harvard Instruments, UK), and their tips were heat polished, typically resulting in resistances of 4–6 MΩ. The standard pipette solution contained (in mM): KCl (140), MgCl_2_ (2), HEPES (10), NaATP (2) GTP (0.1); pH 7.3 with KOH. The standard external solution contained (in mM), NaCl (140), KCl (5), CaCl_2_ (2), MgCl_2_ (1), HEPES (10) and glucose (5); pH 7.3 with NaOH. For recording, mast cells were placed in 35 mm dishes containing standard external solution.

Whole cell currents were recorded using an Axoclamp 200A amplifier (Axon Instruments, Foster City, CA, USA), and currents usually evoked by applying voltage commands to a range of potentials in 10 mV steps from a holding potential of −20 mV. The currents were digitised (sampled at a frequency of 10 kHz), stored on computer and subsequently analysed using pClamp10 software (Axon Instruments). Capacitance transients were minimised using the capacitance neutralisation circuits on the amplifier. Correction for series resistance was not routinely applied. Experiments were performed at 27 °C, temperature being controlled by a Peltier device. Drugs were added directly to the recording chamber as required.

### Confocal microscopy

HEK-293 cells (3×10^5^) were seeded on 25 mm cover glasses and 24 h later dual-transfected with FLAG-tagged K_Ca_3.1 and myc-tagged Orai1 or Orai2 using GeneJuice Transfection Reagent (Novagen). After 24 h, cells were methanol fixed and immunostained. The myc tag was detected with anti-myc 9E10 (gift from ADAS, Nottingham, UK) plus rabbit anti-mouse FITC (F0313, DAKO) alongside anti-mouse IgG1 (X0931, DAKO) isotype control. The FLAG tag was detected by anti-FLAG (F7425, Sigma) plus sheep anti-rabbit RPE (STAR35, AbD Serotec) alongside anti-rabbit IgG (550875, BD Pharmingen) isotype control. Fluorescence microscopy was performed using a Leica TCS SP5 confocal microscope and analysed with ImageJ software. Identical exposures were used for isotype controls.

### Statistical analysis

Data were compared using paired or unpaired *t* test as appropriate. *P* < 0.05 was considered statistically significant.

## Abbreviations

ER: Endoplasmic reticulum
HLMC: Human lung mast cells
LT: Leukotriene
IP_3_: Inositol 1,4,5-triphosphate
